# Retraction: 20-HETE inhibition by HET0016 decreases the blood–brain barrier permeability and brain edema after traumatic brain injury

**DOI:** 10.3389/fnagi.2024.1469759

**Published:** 2024-07-29

**Authors:** Frontiers Editorial Office

The Journal retracts the 2018 article cited above.

Following publication, concerns were raised regarding the integrity of the images in the published figures. Specifically, it was identified that Western blot images in Figures 4A and C had been erroneously duplicated.

The authors were unable to produce the raw data during the investigation, which was conducted in accordance with Frontiers' policies. As a result, the data and conclusions of the article have been deemed unreliable and the article has been retracted. The authors have requested retraction of the article.

